# Evaluation of Sorghum [*Sorghum bicolor* (L.)] Reference Genes in Various Tissues and under Abiotic Stress Conditions for Quantitative Real-Time PCR Data Normalization

**DOI:** 10.3389/fpls.2016.00529

**Published:** 2016-04-25

**Authors:** Palakolanu Sudhakar Reddy, Dumbala Srinivas Reddy, Kaliamoorthy Sivasakthi, Pooja Bhatnagar-Mathur, Vincent Vadez, Kiran K. Sharma

**Affiliations:** International Crops Research Institute for the Semi-Arid TropicsPatancheru, India

**Keywords:** qPCR, RefFinder, *Sorghum bicolor*, gene expression stability, reference gene, normalization

## Abstract

Accurate and reliable gene expression data from qPCR depends on stable reference gene expression for potential gene functional analyses. In this study, 15 reference genes were selected and analyzed in various sample sets including abiotic stress treatments (salt, cold, water stress, heat, and abscisic acid) and tissues (leaves, roots, seedlings, panicle, and mature seeds). Statistical tools, including geNorm, NormFinder and RefFinder, were utilized to assess the suitability of reference genes based on their stability rankings for various sample groups. For abiotic stress, *PP2A* and *CYP* were identified as the most stable genes. In contrast, *EIF4*α was the most stable in the tissue sample set, followed by *PP2A*; *PP2A* was the most stable in all the sample set, followed by *EIF4*α. *GAPDH*, and *UBC1* were the least stably expressed in the tissue and all the sample sets. These results also indicated that the use of two candidate reference genes would be sufficient for the optimization of normalization studies. To further verify the suitability of these genes for use as reference genes, *SbHSF5* and *SbHSF13* gene expression levels were normalized using the most and least stable sorghum reference genes in root and water stressed-leaf tissues of five sorghum varieties. This is the first systematic study of the selection of the most stable reference genes for qPCR-related assays in *Sorghum bicolor* that will potentially benefit future gene expression studies in sorghum and other closely related species.

## Introduction

Sorghum is the fifth most important cereal crop that is widespread in the semi-arid regions of the world with an annual production of 65.5 Mt (FAO, 2011), possesses strong drought-tolerance traits and a high forage value. Sorghum seeds are utilized for both human and animal feed, and cultivars are processed for syrup, sugar, and alcohol. Additionally, sorghum cultivars have great economic potential as they can be valorized for second-generation biofuels to produce environment-friendly energy (Vermerris, 2011; Calviño and Messing, 2012). Sorghum is related to many C4 plants including maize, sugarcane, foxtail millet and switchgrass, which tend to have larger polyploid genomes containing repetitive sequences (Price et al., 2005). In contrast, sorghum is a diploid with a small genome (750 Mbp) and possesses an extraordinary germplasm diversity that greatly aids gene discovery and analysis through comparative and functional genomics, making it highly useful as model cereal for structural and functional genomic studies aimed at improving agronomically important traits (Paterson et al., 2009).

Coupled with species phenotypes, functional genomics can provide important insights into complex biological processes including abiotic stress responses. Functional genomic studies are now being used to identify the roles of regulatory and structural genes. The importance of sorghum functional genomics has increased following the sequencing of its genome (Paterson et al., 2009). Despite a wide range of available experimental approaches for exploring gene expression at the transcriptional level, e.g., northern blotting, ribonuclease protection assays, reverse transcription PCR (RT-PCR), quantitative real-time PCR (qPCR) and DNA microarrays (Valasek and Repa, 2005). Among them, qPCR is the most efficient for quantification of gene expression levels due to its simplicity, sensitivity, accuracy and cost (Wong and Medrano, 2005); however, despite these advantages, the utility of this method is often limited due to the absence of reliable reference genes for qPCR data normalization.

Classically, most of the reference genes fall in the category of housekeeping genes, which have major roles in the maintenance of basic cellular metabolism irrespective of physiological conditions (Bustin, 2002). These reference genes have been widely used in qPCR assays in molecular and functional genomic studies as internal calibrators (Vandesompele et al., 2002; Eisenberg and Levanon, 2013); however, recent studies have shown that these reference genes are often not stably expressed under various experimental conditions (Thellin et al., 1999; Zhu et al., 2013; Gimeno et al., 2014). Consequently, the selection and validation of suitable reference genes that are uniformly and stably expressed across experimental conditions have become imperative (Jian et al., 2008; Li et al., 2012). Various statistical tools, e.g., geNorm, NormFinder, BestKeeper and RefFinder, have been developed to determine reference gene suitability for qPCR data normalization (Vandesompele et al., 2002; Andersen et al., 2004; Pfaffl et al., 2004). Stable reference gene validation has been performed in many plant species, including model species and crop plants such as *Arabidopsis thaliana* (Czechowski et al., 2005; Remans et al., 2008), *Brassica napus* (Yang et al., 2014a; Machado et al., 2015), *Hordium vulgare* (Zmienko et al., 2015), *Fagopyrum tataricum* (Demidenko et al., 2011), *Cajanus cajan* (Sinha et al., 2015), *Oryza sativum* (Jain et al., 2006; Li et al., 2010; Ji et al., 2014), *Arachis hypogaea* (Reddy et al., 2013), *Pennisetum glaucum* (Saha and Blumwald, 2014; Reddy et al., 2015b), *Solanum tuberosum* (Nicot et al., 2005; Mariot et al., 2015), *Glycine max* (Jian et al., 2008; Nakayama et al., 2014), *Saccharum officinarum* (Iskandar et al., 2004; Ling et al., 2015), *Lycopersicon esculentum* (Expósito-Rodríguez et al., 2008), *Triticum aestivum* (Paolacci et al., 2009), and *Zea mays* (Manoli et al., 2012; Lin et al., 2014).

Until now, sorghum qPCR assays were performed using a single reference gene to quantify target gene expression in response to various experimental conditions. Traditional reference genes including *18S RNA, Actin, EIF4*α, *Tubulin*, and *Ubiquitin* have been used as calibrators for quantifying sorghum gene expression in response to abiotic stresses and during plant growth and development (Yang et al., 2004; Jain et al., 2008; Cook et al., 2010; Shen et al., 2010; Wang et al., 2010; Dugas et al., 2011; Ishikawa et al., 2011; Koegel et al., 2013; Li et al., 2013; Gelli et al., 2014; Shakoor et al., 2014; Yang et al., 2014b; Yin et al., 2014; Kebrom and Mullet, 2015; Li et al., 2015; Walder et al., 2015). Nevertheless, the reference genes used in these studies were randomly selected from various sources without any experimental validation. Moreover, single gene quantification qPCR assays are well known to frequently exhibit variability in gene expression under various experimental conditions (Dheda et al., 2005). The Minimum Information for Publication of Quantitative Real-Time PCR Experiments (MIQE) guidelines developed for the proper selection (Bustin et al., 2009) and validation of stable candidate reference genes for qPCR experiments highly recommend averaging data from more than 2 reference genes (Bustin et al., 2010). To date, only one study has evaluated reference genes in virus-infected *Sorghum bicolor* tissues (Zhang et al., 2013), and to the best of our knowledge, there are no systematic studies regarding the selection of suitable stable sorghum reference genes in various tissues and abiotic stress conditions.

This study tested 15 potential candidate reference genes, *Acyl Carrier Protein 2 (ACP2), ADP-Ribosylation Factor (ADPRF), alpha Tubulin (*α*-TUB), beta Tubulin (*β*-TUB), Eukaryotic Initiation Factor 4A-1 (EIF4a), Elongation Factor P (EF-P), Glyceraldehyde 3-phosphate Dehydrogenase (GAPDH), Malonyl CoA-Acyl Carrier Protein (MACP), Malate Dehydrogenase (MDH), Cyclophilin/Peptidylprolyl Isomerase (CYP), Protein Phosphatase 2C (PP2C), 6-Phosphogluconate Dehydrogenase (6PGDH), S-adenosyl methionine Decarboxylase (SAMDC), Serine/threonine-Protein Phosphatase (PP2A), and Ubiquitin Protein 1, Isoform c (UBC1)*. The stabilities of these reference genes were analyzed in sets of samples from treatments with various abiotic stresses (salt, cold, heat, water stress, and ABA stress) and tissues (seedlings, leaves, panicles, roots and mature seeds). Additionally, to verify and support the identification of the best-ranked candidate reference genes, *SbHSF5* and *SbHSF13* gene expression levels were assayed. The stable reference genes identified in this study will be helpful in future qPCR-based molecular and functional studies in sorghum and related species.

## Materials and methods

### Plant materials and abiotic stress treatments

*Sorghum bicolor* genotypes (Parbani Moti, Phule Vasudha (PVS), S35, M35, and BTx623) seeds were collected from the sorghum breeding unit of the International Crops Research Institute for the Semi-Arid Tropics (ICRISAT) in Patancheru, India. Seeds were sown in pots filled with soil mixture (3:2:1 clay:sand:manure) in a glasshouse. Various abiotic stress treatments (salt, cold, heat, water stress, and ABA stresses) were performed and various tissues (seedlings, leaves, panicles, roots, and mature seeds) were sampled according to (Reddy et al., 2015a). Leaf tissues were collected from sorghum cultivars including Parbani Moti, Phule Vasudha, S35, M35, and BTx623 were grown under control conditions and under progressive drought where at normalized transpiration ratio (NTR) of reached at 0.1 (10% of soil moisture remaining in the pot) (Vadez and Sinclair, 2001) then leaf samples were collected. Tissue samples were collected from three individual seedlings for three independent biological replicates, immediately snap frozen in liquid nitrogen and stored at −80°C until RNA extraction.

### Total RNA isolation and cDNA synthesis

Total RNA was extracted from tissues using the Plant RNA mini spin kit (MACHEREY-NAGEL GmbH and Co. KG, Neumann-Neander-Straße 6-8, Duren, Germany) with the in-column DNase I treatment according to the manufacturer's instructions. Sample quantity and purity were determined using a NanoVue Plus Spectrophotometer (GE Healthcare). RNA samples with an OD_260_/OD_280_ absorbance ratio between 1.9 and 2.2 were used for further analysis, and RNA integrity was assessed on 1% denaturing formaldehyde agarose gel. For each sample, 1 μg of total RNA was reverse transcribed using the SuperScript First-Strand Synthesis System for RT-PCR (Invitrogen) and oligo (dT) primers according to manufacturer's instructions. The reverse transcribed cDNAs were then diluted 1:12 with nuclease-free water and used for qPCR analysis.

### Candidate reference gene selection and qPCR primer design

Fifteen candidate reference genes were identified from the literature and the sequences of their corresponding homologs were extracted from the NCBI and the Phytozome databases (Table 1). The selected candidate reference genes included *GAPDH, UBC1, MACP, ACP2*, α*-TUB, EF-P, 6PGDH, SAMDC, CYP, MDH, ADPRF*, β*-TUB, PP2A, EIF4*α, and *PP2C* that were earlier reported to be stably expressed in monocots such as rice, pearl millet, barley, foxtail millet, wheat, and maize (Jain et al., 2006; Paolacci et al., 2009; Manoli et al., 2012; Kumar et al., 2013; Reddy et al., 2015b; Zmienko et al., 2015). Primers were designed using the Primer3 software (Untergasser et al., 2012) with the following parameters: 59–62°C annealing temperature, 20–22 bp primer length, 45–55% GC contents, and 90–150 bp amplicon length (Table 1). Pooled and diluted cDNA samples were used for qPCR and 2% agarose gel electrophoresis was used to check primer pair specificity prior to sequencing. The sequences amplified by each primer combination were compared with GenBank sequences using the BLASTN and BLASTX algorithms to verify amplicon specificity.

**Table 1:** **Comprehensive details of the reference genes used for the normalization in Sorghum**.

**S.No**.	**Gene symbol**	**Gene name**	**Accession number**	**Cellular function**	**Primers (F/R) (5′'–3′')**	**Amplicon length (bp)**	**Tm (°C)**	**PCR efficiency**	**Regression coefficient (R2)**
1	*GAPDH*	*Glyceraldehyde 3-phosphate-Dehydrogenase*	XM_002439118	Glycolysis and gluconeogenesis	AAGGCCGGCATTGCTTTGAAT ACATGTGGCAGATCAGGTCGA	107	84.1	1.00	0.998
2	*UBC1*	*Ubiquitin Protein 1 Isoform C*	XM_002452660	Protein degradation	AGAGGCTCATCTTCGCTGGG CTCAGTTAACACGGCCACCAC	120	88.1	0.98	0.994
3	*MACP*	*Malonyl CoA-Acyl Carrier protein*	XM_002465363	Fatty acid biosynthesis	GCATTGAGAACATCGGGGCTT ATGAGTGGAAACTTCGTTCCA	139	82.8	0.98	0.997
4	*ACP2*	*Acyl Carrier Protein-2*	XM_002462703	Fatty acid and Polyketides biosynthesis	ACGAACTTGTTGCGGCAGAAG GAACAAGAAGGGATGCGCTGG	110	84.3	1.00	0.996
5	*α-TUB*	*Alpha –Tubulin*	XM_002466490	Cytoskeleton structure protein	GAGGGTGAGTTCTCTGAGGCC CTCCCTCATCTCCATCCTCGC	103	85.2	1.00	0.997
6	*EF-P*	*Elongation Factor P*	XM_002463651	Peptide bond synthesis	TGAAGCGGGTGAGAAGATTGT AGCCAAATCATACTCGCCCA	114	80.3	1.00	0.999
7	*6PGDH*	*6-Phosphogluconate dehydrogenase*	XM_002449451	Involved in the pentose phosphate pathway	CACACGGAATGGACCAAGCTG AGAGAAATCACCCAGAGCAGCA	91	85.3	0.97	0.997
8	*SAMDC*	*S-adenosylmethionine decarboxylase*	XM_002446695	Synthesis of polyamines	GTGGCGGACTCCTCATCTACC CCAGTTTGCGGTCCTTCACAG	132	85.1	0.98	0.996
9	*CYP*	*Peptidylprolyl Isomerase*	XM_002453800	*Cis-trans* isomerization of prolineimidic peptide bonds	GTATCTGTGCTCGCCGTCTCT TTCACCCAACTCCTCAACCCC	108	81.1	1.01	0.989
10	*MDH*	*Malate dehydrogenase*	XM_002467034	Citric acid cycle and gluconeogenesis	TGCAGTGGTGGTGAATGGAA GCGTCTTCTCTTCCGACAGC	103	82.5	1.01	0.972
11	*ADP-RF*	*ADP-Ribosylation Factor*	XM_002441244	Regulators of vesicular traffic and actin remodeling	GTCTGTCGGATGTGGGGATGT CACAGCACACAGTCGGACATG	136	84.5	0.96	0.998
12	*β-TUB*	*Beta Tubulin*	XM_002439758	Cytoskeleton structure protein	GCGTGTGAGTCATCCGTTCAC CCGCCTCAGTGAACTCCATCT	98	84.3	1.03	0.999
13	*PP2A*	*Serine/threonine-Protein Phosphatase*	XM_002453490	Control the specific dephosphorylation	AACCCGCAAAACCCCAGACTA TACAGGTCGGGCTCATGGAAC	138	82.7	0.97	0.991
14	*EIF4α*	*Eukaryotic Initiation Factor 4A*	XM_002451491	Eukaryotic translation	CAACTTTGTCACCCGCGATGA TCCAGAAACCTTAGCAGCCCA	144	84.8	1.02	0.993
15	*PP2C*	*Protein Phosphatase 2C*	XM_002468507	Signal transduction	GTAACCCTTCCCGCGAAATCC GCGCTACACTTTGCTGCTTTT	140	85.4	0.94	0.992

*Details of candidate reference genes, NCBI accession number, their putative functions, primer sequences, product size and amplicon characteristics, i.e., melting temperature, PCR efficiency and regression coefficient values*.

### Quantitative real-time RT-PCR (qPCR)

The qPCR reactions were performed using a Realplex Real-Time PCR system (Eppendorf, Germany) and SYBR Green mix (Bioline) in 96 well optical reaction plates (Axygen, USA) sealed with ultra-clear sealing film (Platemax). The reactions were performed in a 10 μl total volume containing 5 μl of 2x SensiMix SYBR No ROX mix (Bioline), 400 nM of each primer, 1.0 μl of diluted cDNA and nuclease-free water. The reaction conditions were 95°C for 2 min, followed by 40 cycles of 15 s at 95°C and 30 s at 62°C with fluorescent signal recording. After amplification, melt curves were generated for each reaction to ensure specific amplification. All qPCR reactions, including the non-template control, were performed in biological and technical triplicates. The mean values obtained from the nine values (triplicates of each biological triplicate) were used to calculate the final quantification cycle values (Cq).

### Data analysis

The quantitative cycle (Cq) values were recorded using the RT-PCR system default settings in which the baseline was automatically corrected and threshold values were estimated using the noise band mode. Statistical analysis (mean and CV) of the Cq values was performed using a Microsoft Excel 2010 spreadsheet. PCR efficiencies (*E*) for candidate genes were evaluated using the dilution series method and pooled cDNA samples. The 12-fold diluted pooled cDNA sample was used for 2-fold serial dilutions. Five serially diluted cDNA samples were used as templates for the construction of standard curves for each primer pair using the above PCR composition and conditions. Standard curves were generated using linear regression based on the quantitative cycle (Cq) values for the dilution series. The correlation coefficients (*R*^2^) and slope values were obtained from the standard curves, and the PCR amplification efficiencies (*E*) were calculated according to the following equation: E = (10^−1∕*slope*^-1).

### Statistical tools for normalization

The genEX Professional software geNorm and NormFinder algorithms (MultiD Analyses AB, Sweden) were used to identify and analyze the stably expressed gene (s) in diverse experimental samples. The raw Cq values for each gene were corrected according to their PCR efficiencies and then converted into relative quantities. The mean values for the biological replicates were used as the input data for the geNorm and NormFinder analyses. The geNorm program calculates the candidate gene expression stability (M value) using pairwise comparisons and stepwise exclusion (Vandesompele et al., 2002). Pairwise variation analysis was performed to identify the optimal number of reference genes required for normalization in each sample set using geNorm and qBase plus software (v: 2.4; Biogazelle, Belgium) (Hellemans et al., 2007). NormFinder also measures candidate reference gene expression stability using stepwise exclusion by estimating the intra- and inter-group variation. A low stability value indicates low combined variation and high expression stability (Andersen et al., 2004). RefFinder is a web-based tool (http://www.leonxie.com/referencegene.php) that integrates the four major computational programs currently available [geNorm (Vandesompele et al., 2002), NormFinder (Andersen et al., 2004), BestKeeper (Pfaffl et al., 2004), and the comparative ΔCt method (Silver et al., 2006)], and calculates the geometric mean for comprehensive ranking.

### *SbHSF5* and *SbHSF13* genes expression

Sorghum heat shock transcription factors *SbHSF5* and *SbHSF13* were selected as target genes for validating stabilities of the most and least stable reference genes by quantifying the gene expression levels in various experimental samples. Sample collections and experiments were performed as described above. *SbHSF5* and *SbHSF13* gene expression levels were normalized using the two most stable candidate reference genes, *PP2A* and *EIF4*α, as well as the two least stable reference genes in the all sample group, *UBC1* and *GAPDH*, individually and in combination. For reference gene validations, root tissues of variety Parbani Moti, and leaf tissues of five sorghum varieties under water stress conditions were used for estimating relative expression levels of *SbHSF5* and *SbHSF13* using REST software (Pfaffl et al., 2002).

## Results

### Primer specificity and PCR efficiency calculations

**Figure 1:**
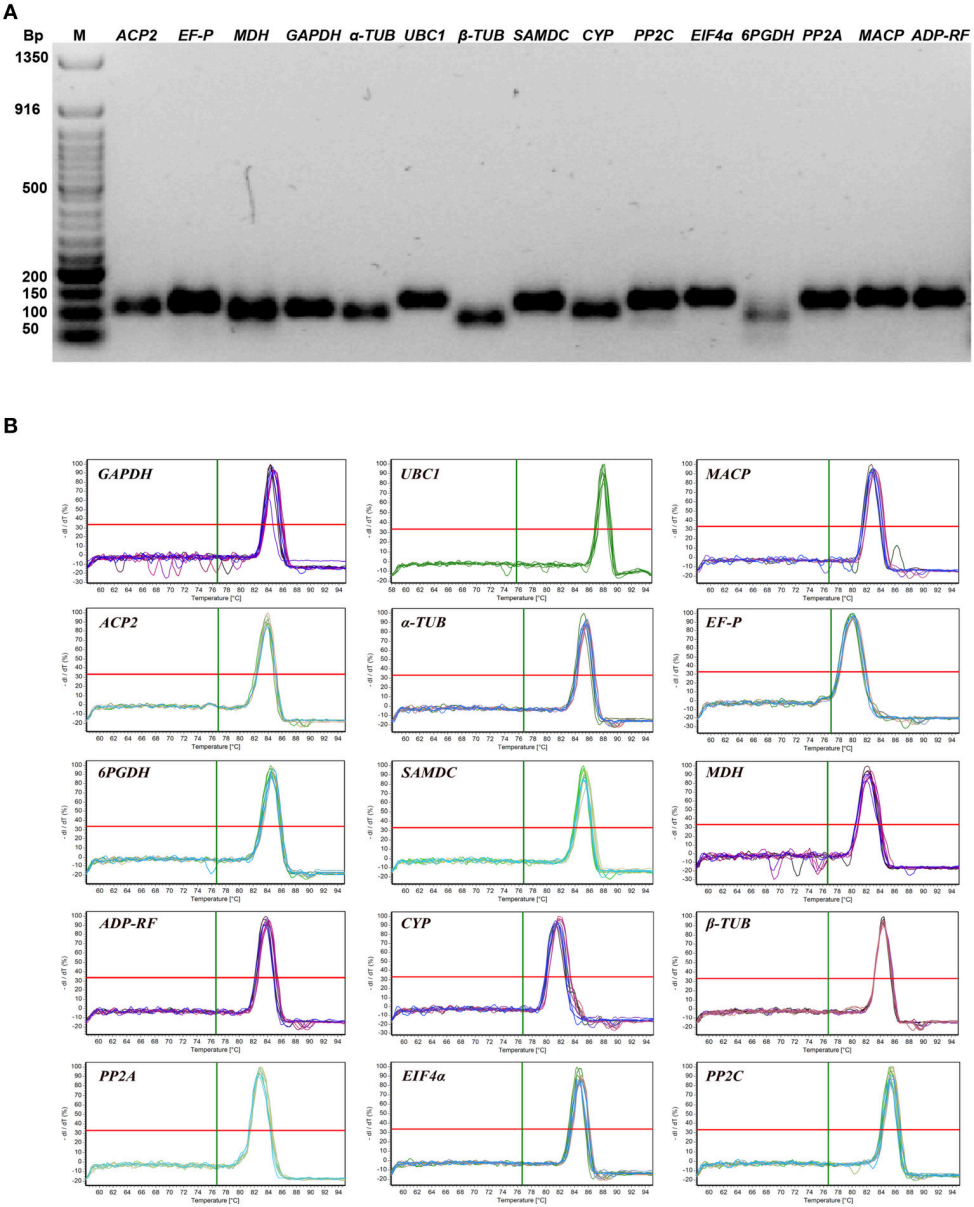
**Specificity of primer pairs for qPCR amplification. (A)** Agarose gel (2.4%) electrophoresis showing PCR products of the expected sizes for 15 candidate genes. M: 50 bp DNA marker (NEB). **(B)** Dissociation curves of 15 candidate reference genes under various experimental conditions, each showing a single peak.

The candidate reference genes selected for this study represent various functional classes and gene families. Primer pairs for 15 candidate reference genes were used for qPCR amplification of sorghum cDNA and yielded single PCR products of the expected sizes (Figure 1A), as well as single melting curve peaks (Figure 1B). The amplification products were sequenced, and verification using the NCBI database BLASTN algorithm revealed 100% matches, demonstrating the gene specificity of the qPCR primer pairs. While the amplification efficiencies (*E*) of the candidate reference genes ranged from 0.94 (*PP2C*) to 1.03 (β*-TUB*), the regression coefficient values (R^2^) ranged from 0.972 (*UBC1*) to 0.999 (*FE-P* and β*-TUB*) (Table 1).

### Sorghum reference gene expression analysis

**Figure 2:**
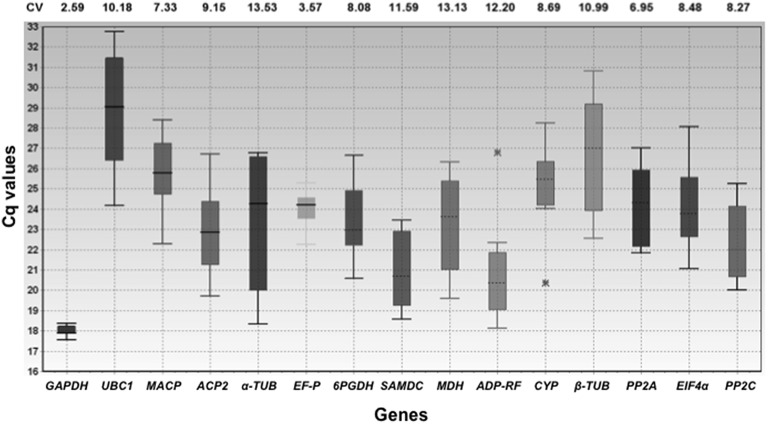
**Expression levels of 15 candidate reference genes in all experimental samples displaying the Ct distribution for each candidate reference gene in all tested samples**. Whiskers represent the maximum and minimum Ct values, and the line across the box indicates the median value, while the asterisks marks outliers. The coefficient of variance (CV) for each gene is given as a percentage. The x-axis represents the genes and the y-axis represents the Cq values.

A total of 15 candidate reference genes were selected for qPCR normalization. A SYBR Green-based qPCR assay was used for transcriptional profiling of the 15 candidate reference genes in 10 samples, including five from abiotic stress conditions (water stress, salt, ABA, cold and heat shock) and five different tissues (leaves, roots, panicles, mature seeds and seedlings). Candidate reference gene transcript levels were determined using the quantification cycle (Cq) values, and these genes varied in their abundance (Figure 2). The mean candidate gene Cq values ranged from 17.70 to 28.92, with most falling between 22 and 25. *GAPDH* exhibited the lowest mean Cq value (17.70), indicating that *GAPDH* was the most highly expressed, while *PP2C, ACP2*, α*-TUB, 6PGDH, MDH, ELFP, EIF4*α, and *PP2A* were moderately expressed (mean Cq 22.31–24.14), whereas β*-TUB* (mean Cq 26.69) and *UBC1* (mean Cq 28.91) were expressed at low levels. Across the tested samples, the *GAPDH* gene showed the least variation in expression levels (coefficient of variation 2.59%), while α*-TUB* was the most variable (13.53%). The degree of variation in reference gene expression across the tested samples is shown in Figure 2. These results indicated that none of the reference gene expression levels were constant and that they varied from one assay to another.

### Reference gene ranking and expression stability analysis

The stability values for each reference gene or combination of reference genes may vary from one experimental set to another. The orders of candidate gene stability ranking for the three sample sets were determined separately to identify the most stable reference genes using three different statistical tools, geNorm, NormFinder and RefFinder.

#### GeNorm analysis

**Figure 3:**
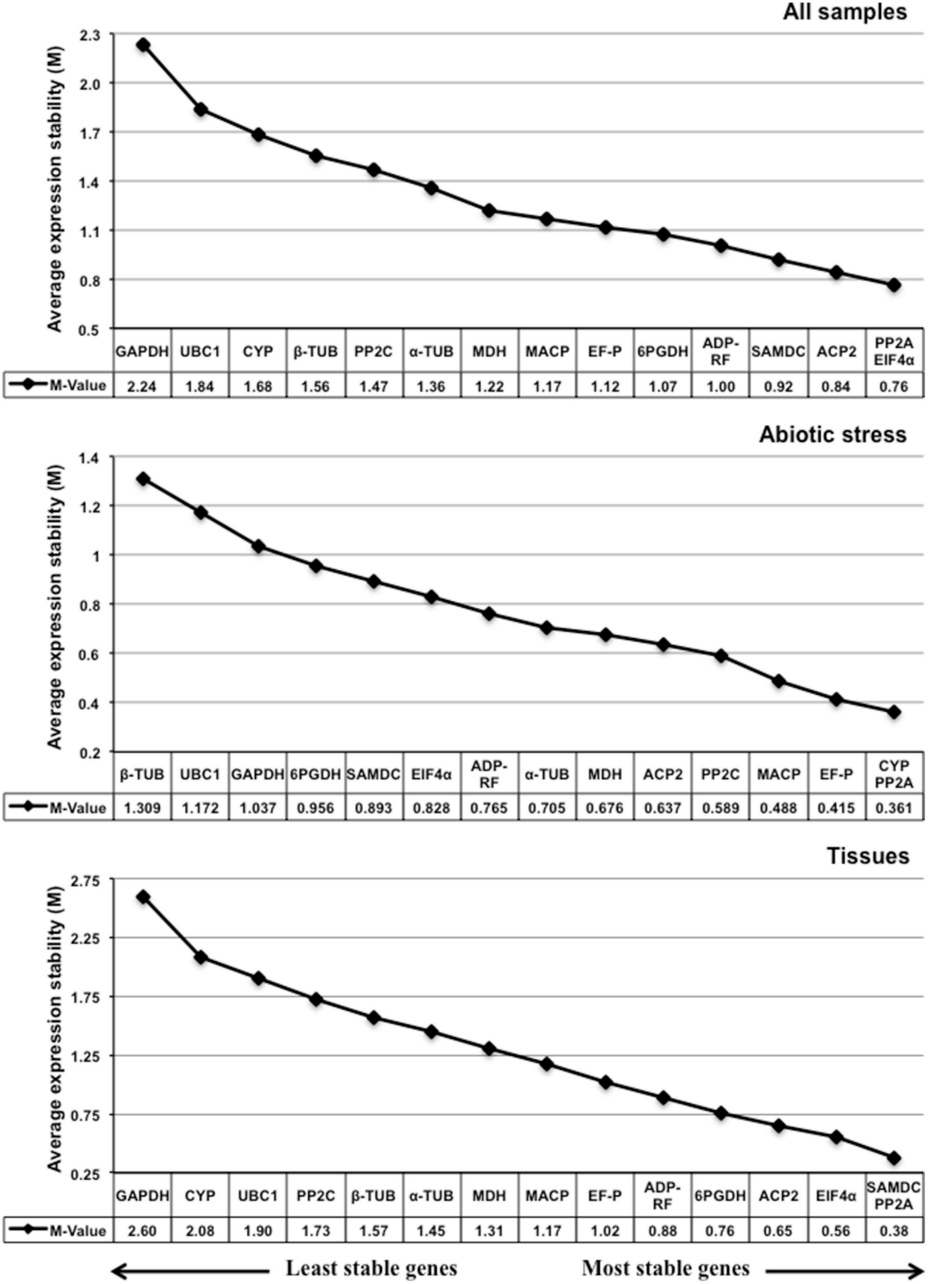
**geNorm expression stability and ranking of the 15 candidate reference genes in various sample sets**. The cutoff M value was set at 1.5; a lower M value indicates greater stability and the largest value indicates the least stable reference gene. The direction of the arrow indicates the most and least stable reference genes. The most stable genes are listed on the right and the least stable genes are listed on the left.

**Figure 4:**
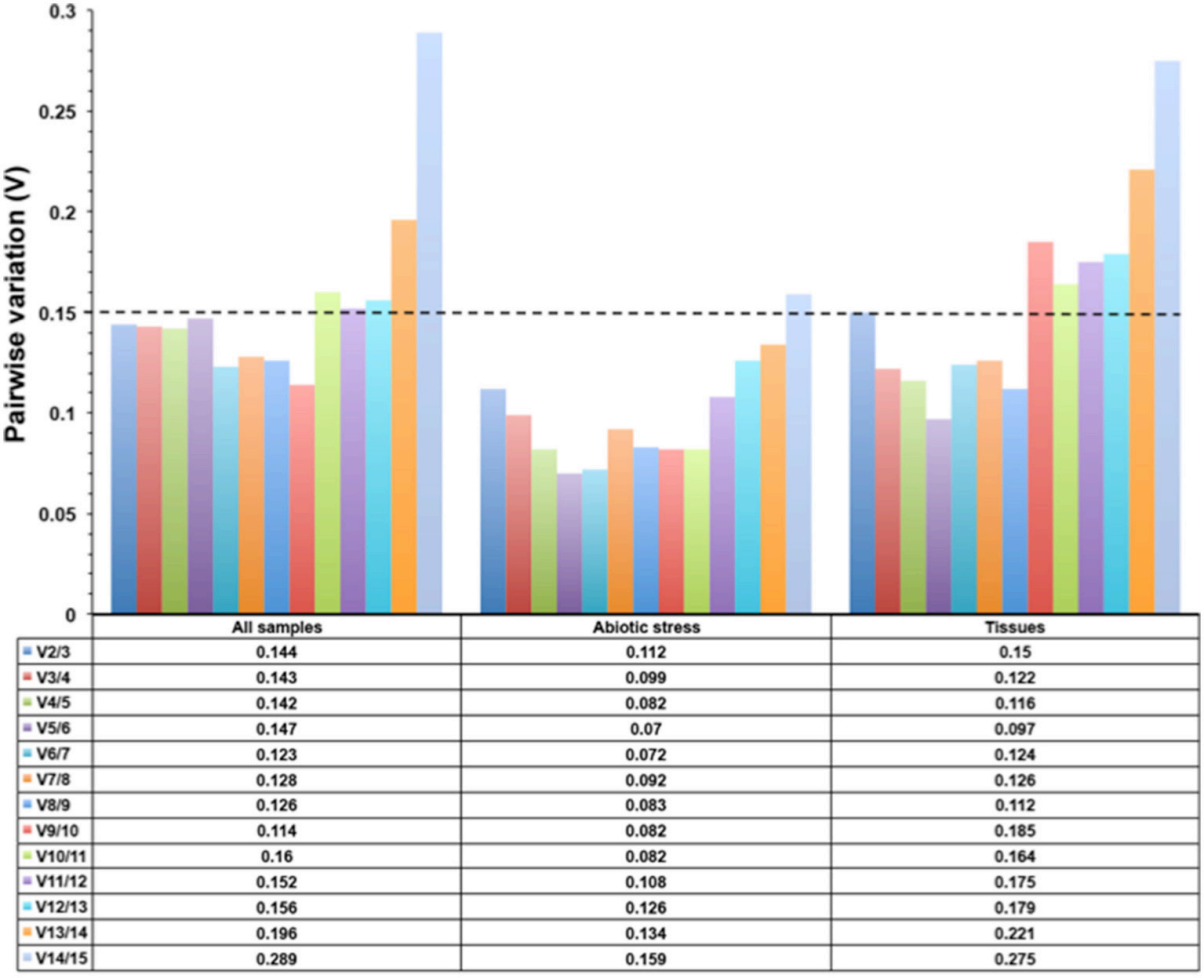
**Optimal number of reference genes required for accurate normalization in all three experimental groups using the geNorm tool**. The pairwise variation (Vn/Vn+1) was calculated by geNorm tool to determine the minimum number of reference genes for accurate normalization in each experimental set. The cutoff value was 0.15, below which additional reference genes are not necessary for gene expression normalization. The dotted line indicates the optimal number of reference genes.

GeNorm software was used to determine the expression stability of the 15 candidate sorghum reference genes; for this analysis, lower M values indicate greater gene expression stability. The software recommends a cutoff M value of 1.5 to identify sets of reference genes that are stably expressed. Figure 3 displays the expression stability rankings for the tested candidate genes; the lowest M value was calculated for the *PP2A* and *EIF4*α pair (*M* = 0.76) and corresponded to the most stable expression in the all sample group, whereas the M values for *GAPDH* and *UBC1* were considerably higher than for the remaining candidate genes (Figure 3). For the abiotic stress treatments, *PP2A* and *CYP* were the most stable reference genes, while *UBC1* and β*-TUB* were the least stable (Figure 3). In the tissue group, *SAMDC* and *PP2A* were the most stable candidate genes while *CYP* and *GAPDH* were the least stable (Figure 3). The optimal numbers of reference genes required for gene expression normalization for the various sample groups were determined using geNorm. As shown in Figure 4, the V2/3 values of all three-sample sets were below the threshold value of 0.15 (0.144 for all pooled samples, 0.112 for the abiotic treatments, and 0.14 for the various tissue sample sets), indicating that two reference genes were sufficient for sorghum gene expression data normalization.

#### NormFinder analysis

**Figure 5:**
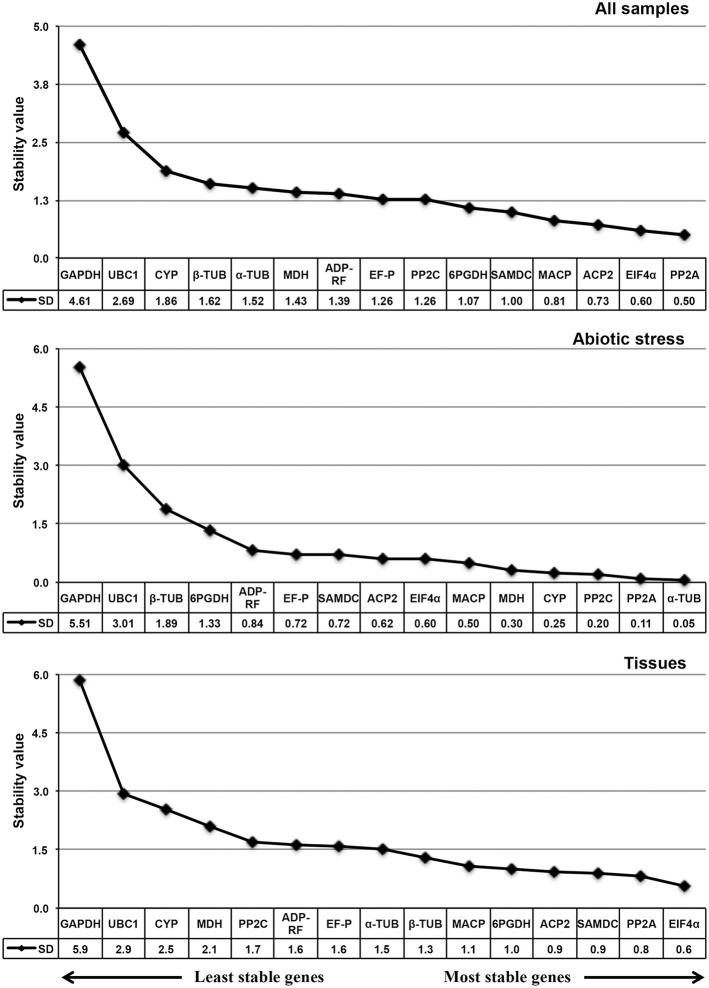
**NormFinder expression stability values and candidate reference gene ranking in *Sorghum bicolor* samples**. Lower values indicate greater stability and larger values indicate the least stable reference genes. The direction of the arrow indicates the most and least stable reference genes. The most stable genes are listed on the right and the least stable are listed on the left.

NormFinder rankings differed slightly from the geNorm rankings (Figure 5). Both tools ranked *PP2A* and *EIF4*α as being the most stable genes in all the sample sets, while *GAPDH* and *UBC1* were the least stable genes (Figure 5); in contrast, α*-TUB* and *PP2A* emerged as the most stably expressed genes in the abiotic stress sample set, while geNorm ranked α*-TUB* eighth (Figure 5). For the tissue sample set, *EIF4*α and *PP2A* reference genes occupied the top positions, while geNorm ranked *EIF4*α third (Figure 5). *GAPDH* and *UBC1* were also the least stable reference genes in the abiotic stress and tissue sample sets (Figure 5).

#### RefFinder analysis

The comprehensive ranking of the candidate reference genes for all three experimental sample sets using RefFinder was highly consistent with the findings of geNorm and NormFinder. RefFinder analysis revealed that *PP2A* and *EIF4*α were the most stable in all the sample set (Table 2). Similarly, *PP2A* and *CYP* were highly stable under abiotic stress conditions, while *EIF4*α and *PP2A* were the two most stable genes for the tissue sample set (Table 2). Comprehensive ranking revealed that *GAPDH* was the least stable gene in the all sample and tissue sample sets, while β*-TUB* was the least stable in the abiotic sample set. *UBC1* was the second most stable gene in all three-sample sets (Table 2). Comprehensive ranking for the individual abiotic stress treatments revealed a pattern similar to that of the abiotic stress sample set in which *PP2A* was second for heat, salt and water stresses. Similarly, *EF-P* remained in the third position for the ABA and clod stresses (Supplementary Table 1).

**Table 2:** **The stability ranking of 15 candidate *Sorghum bicolor* reference genes in three experimental sample sets calculated by delta Ct (DCT), geNorm (GN), NormFinder (NF), BestKeeper (BK) and RefFinder (RF) algorithms**.

**Rank**	**All Samples**					**Abiotic stress**					**Tissues**				
	**DCT**	**GN**	**NF**	**BK**	**RF**	**DCT**	**GN**	**NF**	**BK**	**RF**	**DCT**	**GN**	**NF**	**BK**	**RF**
1	*PP2A*	*PP2A*	*PP2A*	*EF-P*	*PP2A*	*PP2A*	*CYP*	*PP2A*	*GAPDH*	*PP2A*	*EIF4α*	*SAMDC*	*EIF4A*	*EF-P*	*EIF4α*
2	*ACP2*	*EIF4α*	*EIF4α*	*MACP*	*EIF4α*	*CYP*	*PP2A*	*CYP*	*EF-P*	*CYP*	*SAMDC*	*PP2A*	*PP2A*	*PP2C*	*PP2A*
3	*EIF4α*	*ACP2*	*ACP2*	*PP2C*	*ACP2*	*PP2C*	*EF-P*	*α-TUB*	*PP2A*	*EF-P*	*PP2A*	*EIF4α*	*SAMP*	*CYP*	*SAMDC*
4	*MACP*	*SAMDC*	*MACP*	*6PGDH*	*MACP*	*EF-P*	*MACP*	*MDH*	*CYP*	*PP2C*	*ACP2*	*ACP2*	*ACP2*	*6PGDH*	*ACP2*
5	*SAMDC*	*ADP-RF*	*SAMDC*	*CYP*	*EF-P*	*MDH*	*PP2C*	*PP2C*	*EIF4α*	*α-TUB*	*6PGDH*	*6PGDH*	*6PGDH*	*MACP*	*6PGDH*
6	*6PGDH*	*6PGDH*	*6PGDH*	*EIF4α*	*6PGDH*	*α-TUB*	*ACP2*	*EF-P*	*MACP*	*MACP*	*MACP*	*ADP-RF*	*MACP*	*ACP2*	*EF-P*
7	*EF-P*	*EF-P*	*PP2C*	*PP2A*	*SAMDC*	*MACP*	*MDH*	*MACP*	*α-TUB*	*MDH*	*ADP-RF*	*EF-P*	*β-TUB*	*EIF4α*	*MACP*
8	*ADP-RF*	*MACP*	*EF-P*	*ACP2*	*PP2C*	*ACP2*	*α-TUB*	*ACP2*	*ACP2*	*GAPDH*	*EF-P*	*MACP*	*A-TUB*	*UBC1*	*PP2C*
9	*MDH*	*MDH*	*ADP-RF*	*ADP-RF*	*ADP-RF*	*EIF4α*	*ADP-RF*	*EIF4α*	*MDH*	*ACP2*	*β-TUB*	*MDH*	*ELFP*	*PP2A*	*ADP-RF*
10	*PP2C*	*α-TUB*	*MDH*	*SAMDC*	*MDH*	*ADP-RF*	*EIF4α*	*SAMDC*	*ADP-RF*	*EIF4α*	*α-TUB*	*α-TUB*	*ADPRF*	*SAMDC*	*CYP*
11	*α-TUB*	*PP2C*	*α-TUB*	*MDH*	*CYP*	*SAMDC*	*SAMDC*	*ADP-RF*	*PP2C*	*ADP-RF*	*MDH*	*B-TUB*	*PP2C*	*β-TUB*	*β-TUB*
12	*β-TUB*	*β-TUB*	*β-TUB*	*UBC1*	*α-TUB*	*6PGDH*	*6PGDH*	*6PGDH*	*SAMDC*	*SAMDC*	*PP2C*	*PP2C*	*MDH*	*ADP-RF*	*α-TUB*
13	*CYP*	*CYP*	*CYP*	*α-TUB*	*β-TUB*	*GAPDH*	*GAPDH*	*GAPDH*	*6PGDH*	*6PGDH*	*CYP*	*UBC1*	*CYP*	*MDH*	*MDH*
14	*UBC1*	*UBC1*	*UBC1*	*β-TUB*	*UBC1*	*UBC1*	*UBC1*	*UBC1*	*UBC1*	*UBC1*	*UBC1*	*CYP*	*UBC1*	*α-TUB*	*UBC1*
15	*GAPDH*	*GAPDH*	*GAPDH*	*GAPDH*	*GAPDH*	*β-TUB*	*β-TUB*	*β-TUB*	*β-TUB*	*β-TUB*	*GAPDH*	*GAPDH*	*GAPDH*	*GAPDH*	*GAPDH*

### Validation of the best and least ranked sorghum reference genes

**Figure 6:**
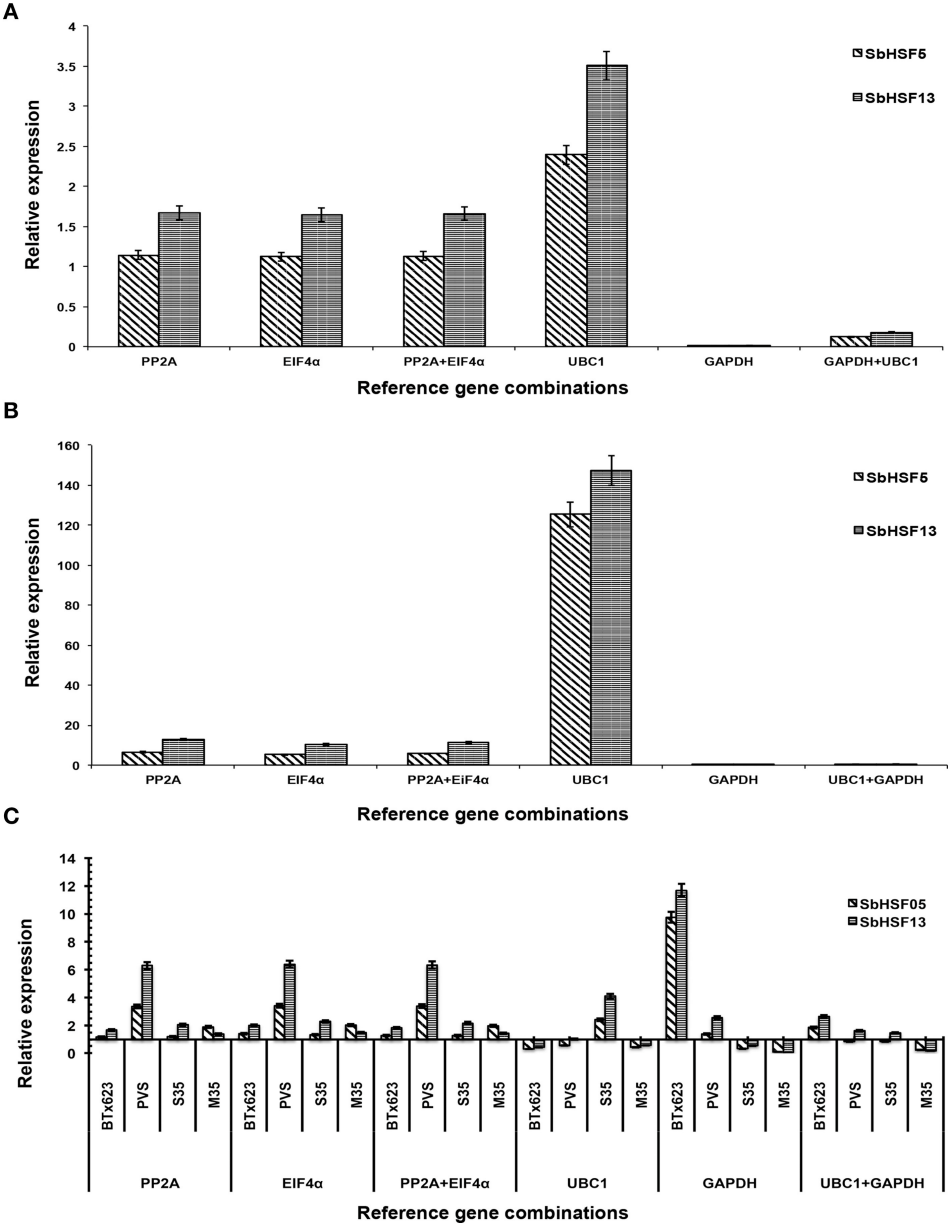
**Relative expression of *SbHSF5* and *SbHSF13* genes in root tissues (A) leaves during water stress treatment (B) selected varieties (C) using different reference genes selected by the RefFinder tool**. *SbHSF5* and *SbHSF13* expression levels were normalized in a single and combined manner with either a stable or unstable reference genes. All samples were analyzed in triplicate, in three independent experiments.

To validate the selected candidate reference genes, the expression patterns of *SbHSF5* and *SbHSF13* were assayed in root tissues, water-stressed samples and different varieties (Figures 6A–C). In sorghum, *SbHSF5* and *SbHSF13* genes have been shown to express in different tissues and induced by abiotic stress treatments (Nagaraju et al., 2015). In this study, the two most stable reference genes from all the sample set, *PP2A* and *EIF4*α, were used for normalization: in roots under normal growth conditions, *SbHSF5* and *SbHSF13* expression were not significantly different. In contrast, the *SbHSF5* and *SbHSF13* expression patterns were very different when *GAPDH* and *UBC1*, the least stable genes from all the sample set, were used for normalization. Normalization with *UBC1* indicated a 2- to 3-fold increase in expression, while normalization with *GAPDH* and *GAPDH*+*UBC1* indicated downregulation of both genes in root tissues under normal conditions (Figure 6A). To further validate the selected reference genes, the transcript levels were quantified in five sorghum varieties under water stress conditions (Figure 6C). For water stress samples, *SbHSF5* and *SbHSF13* expression levels increased1 to 12-fold under water stress conditions when normalized with the two most stable reference genes from all the sample set (*PP2A* and *EIF4*α; Figures 6B,C). As expected, normalization with the least stable genes (*GAPDH* and *UBC1*), either alone or in combination with *UBC1* resulted in discrepancies for expression of both *SbHSF5* and *SbHSF13* gene expression (Figures 6B,C).

## Discussion

Accurate gene expression primarily relies on the stable expression of reference genes under diverse experimental conditions; however, thus far, no single reference gene has been shown to be stably expressed across experimental conditions (Olsvik et al., 2008; Cruz et al., 2009). The stability value of the most stable reference gene or the best combination of genes may vary from one experimental setup to another, meaning that the reference genes used for normalization should be validated under certain experimental conditions by statistically and experimentally. In the present study, 15 candidate reference genes were evaluated for expression stability among diverse experimental samples including various tissues and abiotic stress conditions. Expression analysis revealed that all 15 candidate reference genes varied significantly across the 10 tested samples; therefore, reference genes for optimal normalization were chosen from a set of candidate genes for each experiment either alone or in combination. To determine the comprehensive ranking of candidate reference genes in a sample set, we also used RefFinder, which considers the results of the four algorithms (geNorm, NormFinder, BestKeeper and the ΔCq method) together. Based on their stability rankings, various candidate genes have been proposed for their suitability as reference genes depending on the experimental conditions, implying that the reference genes used for abiotic stress studies in sorghum might not be suitable for gene expression analysis across genotypes and species.

In all the sample set, all three algorithms identified two reference genes, *PP2A* and *EIF4*α, as the two most stable genes. The pairwise variation calculated by geNorm also indicated that the use of two genes would be sufficient for normalization (Figure 4). Based on these results, it is inferred that *PP2A* and *EIF4*α would be appropriate for qPCR data normalization for the sorghum all sample set. For the abiotic stress treatment sample set, the pairwise variation indicated that the top ranking two genes (*PP2A* and *CYP*) could be used for normalization (Figure 4). Similarly, *PP2A* and *EIF4*α would be sufficient for the normalization of the tissue sample set data (Figure 4). *PP2A* was identified as one of the two most stable genes in all three studied sample sets (Table 2). This result is in agreement with previous studies of maturing *B. napus* embryos (Chen et al., 2010), multiple stress conditions (Wang et al., 2014), development and under various environmental conditions in *A. thaliana* (Czechowski et al., 2005) and *Caragana intermedia* (Zhu et al., 2013), as well as for virus-infected *Nicotiana benthamiana* (Liu et al., 2012) and *Z. mays* (Zhang et al., 2013). Furthermore, *PP2A* was the most stably expressed gene among various Striga life stages (Fernández-Aparicio et al., 2013). *EIF4*α was second most stably expressed gene in the all sample and tissue sample sets (Table 2). Similar results were observed in *Carica papaya*, where *EIF4*α expression was stable under most of the experimental conditions tested (Zhu et al., 2012). *EIF4*α was also reported as being a stably expressed gene in sexual and apomictic *Brachiaria brizantha* (Silveira et al., 2009), and in sexual and apomictic accessions of *Cenchrus ciliaris* (Simon et al., 2013). In the present study, *CYP* was the third most stable gene; it was stably expressed under abiotic stress treatments and in the tissue sample set (Table 2). The results obtained in the present study are in agreement with our previous studies in peanuts, where *CYP* was the most stably expressed gene during vegetative stages and under abiotic stress conditions (Reddy et al., 2013) and was also stably expressed in various tissues of *G. max* (Jian et al., 2008), *Vicia faba* (Gutierrez et al., 2011) and salt-stressed *S. tuberosusm* (Nicot et al., 2005). The *CYP* gene was moderately stable in developing and germinating *G. max* seeds (Li et al., 2012) and in bananas under various experimental conditions (Chen et al., 2011). In contrast, our recent study in *Cicer arietinum* indicated that *CYP* was the least stably expressed gene under various experimental conditions (Reddy et al., 2016), consistent with a previous study of grapevine berry development (Reid et al., 2006). In conclusion, *PP2A, EIF4*α, and *CYP* are recommended as suitable reference genes for the qPCR-based normalization of gene expression in sorghum under abiotic stress and in various tissues.

To further validate the candidate reference genes selected from the RefFinder comprehensive ranking, the expression levels of two sorghum heat shock transcription factors, *SbHSF5* and *SbHSF13*, were assessed. Plant heat shock transcription factors play major roles in higher plants under various abiotic stresses. In sorghum, *SbHSF5* and *SbHSF13* are expressed under abiotic stress conditions: *SbHSF5* is moderately expressed under cold stress and upregulated under drought stress, and *SbHSF13* is moderately expressed under cold and heat stress and upregulated under drought and high salinity stress (Nagaraju et al., 2015). In the present study, *SbHSF5* and *SbHSF13* expression levels were upregulated under water stress conditions and no significant changes were observed in root tissue gene expression levels when normalized to the most stable reference genes, *PP2A* and *EIF4*α (Figures 6A,B). While, *SbHSF5* and *SbHSF13* genes significantly upregulated under water stress conditions in leaf tissues of all five sorghum genotypes with relatively higher expression when normalized with the sable reference genes (*PP2A* and *EIF4*α), the normalization was obscured when least stable reference gene (s) (*UBC1* and *GAPDH*) were used (Figures 6B,C). The upregulation of *SbHSF5* and *SbHSF13* under this study is in agreement with another recent study in sorghum (Nagaraju et al., 2015) confirming the stability validation of the selected candidate reference genes and demonstrated the disadvantages of using unstable reference genes for normalization.

## Conclusion

This study reports on the selection and validation of stable reference genes for qPCR-based gene expression studies in sorghum in response to various abiotic stresses and in different tissues. Three major statistical tools, geNorm, NormFinder and RefFinder, were used to analyze the suitability of reference genes. Some slight differences were observed between the statistical tools with regards to the selected stable reference genes. Overall, *PP2A* and *EIF4*α are the most stable candidate reference genes, and *UBC1* and *GAPDH* were found to be least stable. The use of two reference genes would be optimal for the normalization of sorghum gene expression levels in various tissues and abiotic stress conditions. The most and least stable reference genes were further validated for their suitability as reference genes by normalizing *SbHSF5* and *SbHSF13* expression levels under various experimental conditions across five genotypes. This work will benefit future studies of gene expression in related experiments using different tissues and abiotic stress conditions in *S. bicolor* and related crops.

## Author contributions

PS, KS, and VV designed the experiments. PS, DS, SK, and PB performed the experiments and data analysis. PS, DS and KS drafted the manuscript.

## Funding

Financial support to PS through the INSPIRE Faculty Award (IFA11-LSPA-06) and the Young Scientist Scheme SB/YS/LS-12/2013 provided by the Department of Science and Technology, Government of India, New Delhi is gratefully acknowledged. This work was performed as part of the CGIAR Research Program on Dryland Cereals.

### Conflict of interest statement

The authors declare that the research was conducted in the absence of any commercial or financial relationships that could be construed as a potential conflict of interest.

## Abbreviations

MACP: Malonyl CoA-Acyl Carrier protein
6PGDH: 6-Phosphogluconate dehydrogenase
SAMDC: S-adenosylmethionine decarboxylase
CYP: Cyclophilin/Peptidylprolyl Isomerase
PP2A: Serine/threonine-Protein Phosphatase 2A
qPCR: Quantitative Real-Time Polymerase Chain Reaction.
